# The National Israeli Field Hospital in Ukraine: Innovative adaptation to a unique scenario

**DOI:** 10.7189/jogh.12.03078

**Published:** 2022-10-16

**Authors:** Elhanan Bar-On, Asaf Vivante, David Dagan, Yoel Har Even, Galia Barkai, Ariel Furer, Michael Bass, Michal Kirshenbaum, Omer Niv, Leonid Barski, Adam Lee Goldstein, Rami Sagi, Danny Moshayov, Shani Brosh, Michal Mekel, Eldad Katorza, Yitshak Kreiss

**Affiliations:** 1 Sheba Medical Center, Tel Hashomer, Israel; 2 Sackler School of Medicine, Tel Aviv University, Israel; 3 Israel Ministry of Health; 4 Faculty of Medicine, Hebrew University of Jerusalem, Israel; 5 Maccabi Health Services, Israel; 6 Schneider Children's Medical Center of Israel; 7 Soroka University Medical Center, Beer Sheva, Israel; 8 Wolfson Medical Center, Holon, Israel; 9 HMO - Meuhedet, Israel; 10 Carmel Medical Center, Haifa, Israel; 11 Rambam Medical Center, Haifa, Israel; 12 Technion - Institute of Technology, Haifa, Israel

On February 24, 2022 hostilities broke out in Ukraine resulting in a full-scale humanitarian crisis with thousands of civilians injured, twelve million persons displaced – seven million internally displaced and five million refugees fleeing the country. The crisis led to a severe disruption of routine medical services. These circumstances, combining warfare and refugees, pose major challenges to the international aid community: Treating the wounded, addressing the medical and psychosocial needs of the displaced populations, meeting routine medical needs of under-served local populations, and strengthening resilience through capacity building of the disrupted local healthcare system [1,2].

## MISSION

The State of Israel, which in all its past humanitarian missions deployed military field hospitals [3-5], decided, for the first time, to deploy a civilian field hospital. To our knowledge, it was the only state-level foreign field hospital deployed in Ukraine. This unique mission required addressing immediate challenges in the pre-deployment phase: Understanding the exact medical needs, defining essential logistic and security requirements, coordinating with the local government, and designing the field hospital accordingly. A Rapid Assessment Team was dispatched and worked in close collaboration with The Israeli Ministry of Foreign Affairs team on the ground and with The Ukrainian health and local authorities, subsequently discovering that the needs were different from those we expected. We initially considered deploying in Poland, adjacent to the large refugee camps. However, we found that most refugees stayed in the camps for only 2-3 days before continuing to various destinations and those requiring medical treatment were treated in Polish hospitals. We also became aware that inside Ukraine, although the war casualties were being treated in Ukrainian military hospitals in the combat zones, there was a severe disruption of routine medical services and interruption of most elective activity, leaving the local population as well as the refugees deprived of basic care.

**Figure:**
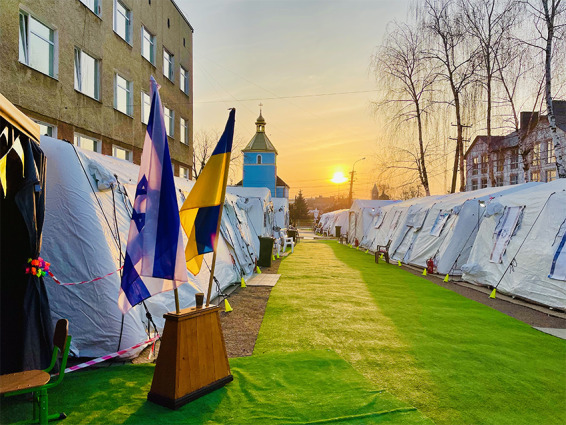
Photo: The Israeli field hospital in Ukraine. Source: Sheba Medical Center, used with permission.

We were, therefore, faced with a dilemma regarding site selection for the field hospital: while it was evident that the main medical needs were inside Ukraine, we had to consider the security risks of operating in a country at war. Although the main battlegrounds were in the east of the country, we were aware of sporadic missile firing in our planned deployment area. This necessitated taking specific security measures and we therefore planned our deployment accordingly, based on our past experience in similar situations in Israel.

The location ultimately selected for the field hospital was the town of Mostyska, on the main refugee route, 15 km east of the Medyka crossing in an area not directly affected by the combat. This enabled easy access to the hospital while providing relative safety for the patients and staff. In retrospect, we feel that despite the increased security risk, deploying the hospital inside Ukraine, amongst the needy population was the right decision.

**Figure 1:**
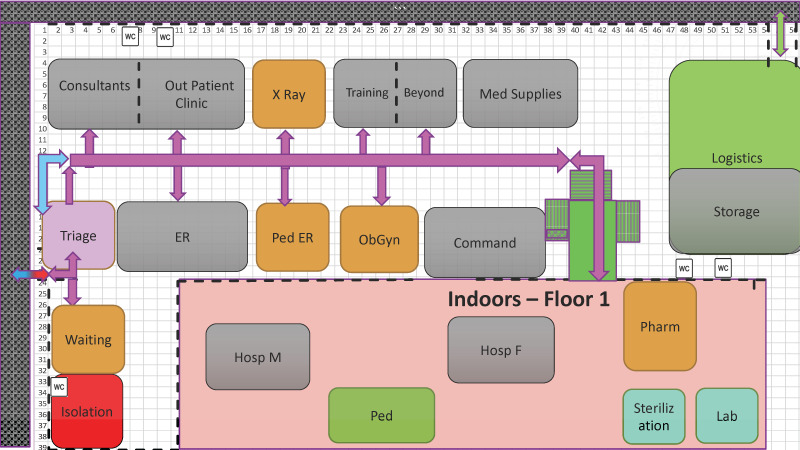
Field Hospital layout and patient flow (Source: Sheba medical Center, used with permission).

The hospital was established in a school complex with a tented facility housing out-patient activities (**Figure 1**). Hospitalization wards, pharmacy, laboratory and staff accommodation were housed in converted classrooms inside the school building, providing increased protection as well as a cellar serving as an air-raid shelter during the daily siren alerts. Functional units of the hospital included triage, emergency department, outpatient clinics, paediatrics, obstetrics and gynaecology, surgery and orthopaedics, a mental-health team composed of psychiatrists and social workers, and auxiliary services – imaging, laboratory, pharmacy and medical logistics as well as an advanced Information technology (IT) and medical records system. The hospital was completely digitalized and was integrated into the IT system of Sheba Medical Center - the dispatching hospital in Israel. For this mission we also added an innovative telemedicine unit (Sheba Beyond) and a special team for training healthcare professionals. As part of our deployment policy [5], we established a collaboration system with the local leadership for referral, evacuation and coordination of surgical activity, to be carried out in the adjacent local hospital by mixed surgical teams.

The hospital commenced operation on March 22, 2022 and was operational for six weeks. During this period 6161 patients were treated in the hospital of which 954 were children.

The conditions treated ranged from chronic non-communicable diseases such as hypertension, diabetes and osteoarthritis, to acute and emergency conditions including, among others: infections, myocardial infarction, hypertensive crisis, acute appendicitis, acute cholecystitis, perforated duodenal ulcers and fractures. 65 patients were hospitalized and 59 patients underwent surgery. 103 patients had consultations utilizing telemedicine technologies. 995 patients had laboratory tests, and 846 patients underwent imaging studies. Seven patients were brought to Israel for sophisticated care which was unavailable in the field hospital or in local health facilities. The hospital was dismantled after six weeks of operation as there was a decline in the refugee flow and it was felt that the acute phase needs had been addressed, as well as for budgetary reasons. However, we maintained contact with local medical facilities for continued support and capacity building.

## INSIGHTS

The scenario of operating in a war zone with a large refugee population, and establishing a field hospital for the first time by a civilian entity, posed unique challenges and necessitated constant adaptations during both the planning and the operational stages. Several important lessons were learned during our deployment: contrary to our initial expectations, despite the operation being in a war zone, the main necessity encountered was delivery of primary care to both the refugee and local population, due to the compromised medical system. Capabilities were enhanced accordingly, shifting the emphasis from surgery of war wounds to ambulatory routine medicine, increasing the number of primary care physicians in the team, and task shifting to the areas of increased patient load. In order to maximize access to advanced secondary care, we implemented, for the first time, large-scale telemedicine technologies. This special platform (Sheba Beyond), was based on the capabilities developed during the COVID-19 pandemic which brought with it major advances in telemedicine techniques and a wide range of opportunities for delivering remote care [6]. These were used widely during our deployment, enabling consultation with the most experienced specialists in Israel.

**Figure 2:**
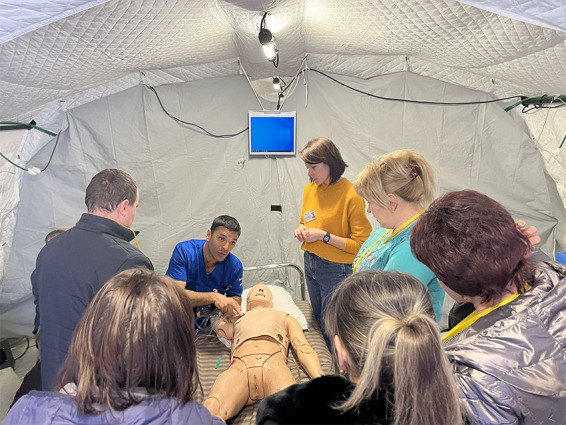
Simulation training of Ukrainian medical staff (Source: Sheba medical Center, used with permission).

An important component of humanitarian missions is increasing the impact in the post deployment phase by continued local capacity building. Therefore, our capacity-building program was given high priority during the mission. It included bringing dedicated staff and mannequins from Israel's National Center for Medical Simulation (MSR), and delivering structured training courses (**Figure 2**). In addition, clinical training was provided in the clinics, wards and joint surgical activities as well as in telemedicine techniques. During our deployment 796 Ukrainian medical personnel received training in a wide variety of medical and psychosocial fields. In addition, hardware was distributed, enabling the continued use of telemedicine by the local teams, thus increasing the access of deprived populations to high-level medical care. We believe that this capacity-building effort should not be limited to the period of deployment but rather, use the acute phase deployment as an opportunity to establish a dialogue with the local healthcare community for continued collaboration and support. At the time of writing, several continued collaboration projects with Ukrainian Medical centres are being planned.

Besides the field hospital activity of treating patients and training medical staff, we believe that “not everything that counts can be counted”. Throughout the mission, we felt a sense of trust and gratitude from all those we encountered – patients, medical staff and administrators. Once again, we learned that dispatching a team delivering medical care and extending a helping hand in time of need, especially when done by a national entity, contributes greatly to building the people's faith, hope and resilience during the crisis, and has a critical role in the recovery effort. The dilemma of dispatching a civilian team to a war zone and investing resources in this endeavour will always exist, however, we feel that providing hope to a nation in need is part of our duty as humanitarians and healthcare providers and we hope the international health community will devote the necessary resources to this important cause.
